# Sick leave and healthcare utilisation in women reporting pregnancy related low back pain and/or pelvic girdle pain at 14 months postpartum

**DOI:** 10.1186/s12998-016-0088-9

**Published:** 2016-02-15

**Authors:** Cecilia Bergström, Margareta Persson, Ingrid Mogren

**Affiliations:** Department of Clinical Sciences, Obstetrics and Gynecology, Umeå University, Umeå, Sweden; Department of Nursing, Umeå University, Umeå, Sweden

**Keywords:** Pelvic girdle pain, Pregnancy related low back pain, Sick leave, Healthcare utilisation, Postpartum period, Female, Pregnancy, Pregnancy complications, Cohort studies

## Abstract

**Background:**

Pregnancy related low back pain (PLBP) and pelvic girdle pain (PGP) are considered common complications of pregnancy. The long-term consequences for women with persistent PLBP/PGP postpartum are under-investigated. The main objective was to investigate the prevalence, pattern and degree of sick leave as well as healthcare utilisation and its perceived effect in women with persistent PLBP/PGP at 12 months postpartum.

**Method:**

This is a follow-up study of a cohort involving of a sample of women, who delivered from January 1^st^ 2002 to April 30^th^ in 2002 at Umeå University Hospital and Sunderby Hospital, and who reported PLBP/PGP during pregnancy. A total of 639 women were followed-up by a second questionnaire (Q2) at approximately 6 months postpartum. Women with persistent PLBP/PGP at the second questionnaire (*N =* 200) were sent a third questionnaire (Q3) at approximately 12 months postpartum.

**Results:**

The final study sample consisted of 176 women reporting PLBP/PGP postpartum where *N* = 34 (19.3 %) reported ‘no’ pain, *N* = 115 (65.3 %) ‘recurrent’ pain, and *N* = 27 (15.3 %) ‘continuous’ pain. The vast majority (92.4 %) of women reported that they had neither been on sick leave nor sought any healthcare services (64.1 %) during the past 6 months at Q3. Women with ‘continuous’ pain at Q3 reported a higher extent of sick leave and healthcare seeking behaviour compared to women with ‘recurrent’ pain at Q3. Most women with persistent PLBP/PGP had been on sick leave on a full-time basis. The most commonly sought healthcare was physiotherapy, followed by consultation with a medical doctor, acupuncture and chiropractic.

**Conclusion:**

Most women did not report any sick leave or sought any healthcare due to PLBP/PGP the past 6 months at Q3. However, women with ‘continuous’ PLBP/PGP 14 months postpartum did report a higher prevalence and degree of sick leave and sought healthcare to a higher extent compared to women with ‘recurrent’ PLBP/PGP at Q3. Women with more pronounced symptoms might constitute a specific subgroup of patients with a less favourable long-term outcome, thus PLBP/PGP needs to be addressed early in pregnancy to reduce both individual suffering and the risk of transition into chronicity.

## Background

Pregnancy related low back pain (PLBP) and pelvic girdle pain (PGP) are common complications of pregnancy and represent a significant health problem among women both during and after pregnancy [1–3]. PLBP resembles low back pain (LBP) that occurs in a non-pregnant state while PGP is described as pain between the two posterior iliac crests in the proximity of the sacroiliac joints (SI-joints), the gluteal folds and with or without pain in the symphysis pubis and/or down the posterior thigh [4]. The prevalence of PLBP/PGP among pregnant women ranges from 4 to 76.4 % depending on definition used [5].

PLBP/PGP is not a self-limiting condition in some women [1]. Research has shown that among women developing PLBP/PGP during pregnancy about 80 % of women report mild complaints of PLBP/PGP postpartum, whereas 13 % of women report moderate and 7 % have very serious complaints [6]. In addition, women suffering from PGP postpartum seem to have a higher prevalence of depressive symptoms compared to women without postpartum PGP [7]. Predictors of poor outcome postpartum have shown to be: previous LBP, high levels of pain postpartum, high body mass index (BMI), high maternal age, hypermobility, physical strenuous work situation and low job satisfaction [1, 8–13]. Furthermore, there is an increased likelihood of reporting poorer health status in women reporting continuous pain postpartum [1]. Consequently, women with recurrent or continuous pain postpartum may have a poor prognosis in regard to future sick leave and disability.

The World Health Organization (WHO) considers musculoskeletal conditions to be the second greatest cause of years lived with disability (YLD), where low back pain ranks number one of the top 10 leading causes of global YLD [14]. Spine-related problems constitute large individual and societal costs as a result of chronic musculoskeletal pain. In Sweden, the cost has been estimated to SEK 87.5 billion (EUR 8.6 million), where over 90 % of the total costs are associated with indirect costs due to sickness absence and disability pension [15]. In addition, international studies have reported a higher utilisation of health services with regard to chronic pain [16–18].

Even though not all women with PLBP/PGP during pregnancy transition into a more chronic state postpartum, women with persistent problems are an understudied group of patients and relatively few studies have a longer follow-up time of more than 3 months [2, 3, 19–22]. Moreover, these women may constitute a specific subgroup of patients within the heterogeneous back pain population consuming a significant part of the allocated resources provided by the social security and healthcare systems. Therefore we wanted to investigate sick leave and healthcare utilisation in women with ‘recurrent’ or ‘continuous’ PLBP/PGP at approximately 12 months postpartum. More specifically, we wanted to determine the prevalence, pattern and degree of sick leave in women with ‘recurrent’ and ‘continuous’ PLBP/PGP during pregnancy, at 6 and 12 months postpartum. In addition, we wanted to investigate what type of healthcare had been sought the past 6 months at the 12 months postpartum follow-up and its perceived effect on symptoms.

## Method

### Design

This is a follow-up study that is part of a longitudinal cohort of pregnant women reporting persistent PLBP/PGP at 12 months postpartum and the project has been described in detail elsewhere [1]. Briefly, this is a longitudinal study consisting of a sample of women who delivered from January 1^st^ 2002 to April 30^th^ in 2002 at Umeå University Hospital (UUH) and Sunderby Hospital (SH), the largest hospitals situated in the two most northern counties of Sweden.

### Data collection

Baseline data were collected through a questionnaire (Q1) in close proximity after delivery and women reporting PLBP/PGP at baseline (Q1) were thereafter invited to complete a second questionnaire (Q2) at 6 months postpartum. A third questionnaire (Q3) was distributed approximately 12 months postpartum to all women reporting persistent ‘recurrent’ or ‘continuous’ pain at Q2.

All questionnaires (Q1, Q2 and Q3) included issues such as persistence or remission of symptoms, use of medical services, family situation, SRH, sick leave, sexual life, physical activity, oral contraception and breastfeeding among other variables. Relevant background variables from Q1-Q3 for the research question in this study are presented in this paper.

### Study participants

**Fig. 1:**
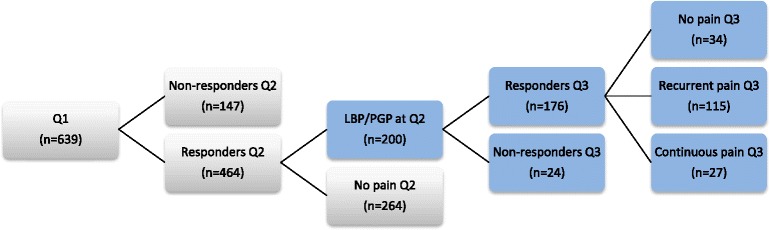
Overview of the entire cohort

Detailed description of inclusion criteria and procedure are presented in another publication from this cohort [1]. The final study sample responding to Q3 comprised of 176 women (88.0 %) out of the 200 women who reported ‘recurrent’ or ‘continuous’ pain at 6 months postpartum (Q2). An overview of the entire cohort is illustrated in Fig. 1.

### Validity of data

The validity of the data collected at Q1 has previously been discussed at length [23]. In brief, respondents and non-respondents did not differ concerning maternal age, gestational age, birth weight, mode of delivery, total experience of delivery, epidural or spinal anaesthesia during delivery, and pre-pregnancy or end-pregnancy BMI. Moreover, no difference was found between respondents and non-respondents in regard to baseline variables except for smoking and maternal age at first delivery. Consequently, the conclusion was that the data collected through Q1 seem to be representative for Swedish women with persistent LBP and/or PGP postpartum [23]. Questions included in Q2 and Q3 were similar to those in Q1.

### Definitions of variables

PLBP/PGP at Q3 was defined when the woman reported ‘recurrent’ pain or ‘continuous’ pain due to PLBP/PGP when responding to Q3. The response alternatives to the question: ‘Do you experience low back pain or pelvic pain right now?’ were ‘yes, recurrent pain’, ‘yes, continuous pain’ and ‘no’ pain. In addition, a pain drawing was included where marking of the affected area could be indicated. Women who reported a specific time point in Q3, at which PLBP/PGP had ceased (even though reporting ‘recurrent’ pain), were allocated to the ‘no’ pain group.

*Sick leave*. Information about sick leave was self-reported. The participants were asked if they had been on sick leave at all three time points (Q1-Q3) due to PLBP/PGP (response alternatives ‘yes’ or ‘no’). In addition, at each time point (Q1 – Q3) they were asked to report how many weeks and to what degree (response options: full-time, part-time or both full- and part-time) they had been on sick leave. The participants were also asked to what degree they had been on sick leave during the pregnancy (Q1) and during the past 6 months at Q2 and Q3 due to PLBP/PGP. The response alternatives were ‘full-time’ or ‘part-time’. Long-term sickness absence is often defined as more than 30 days [24, 25] as sick leave of less than 30 days is found to be a predictor of short-term recovery [26]. Consequently, sick leave was dichotomized into less or more than 4 weeks of self-reported sick leave, irrespective of full-time or part-time sick leave.

*Healthcare services* were defined as healthcare provided by a practitioner in allopathic medicine or complementary and alternative medicine for the PLBP/PGP. Participants were asked to recall the total numbers of visits to a healthcare provider during the past 6 months at Q2 and Q3 and also, the perceived effect that a particular treatment had on their PLBP/PGP symptoms (response alternatives ‘no effect’, ‘some effect’, ‘good effect’).

*Pre*-*pregnancy* weight was defined as self-reported weight before the actual pregnancy and *end-pregnancy* weight was defined as reported weight before delivery. Self-reported weight was also asked for in kg at Q2 and Q3. *Height* was given in centimetres (cm). *Body Mass Index (BMI)* was defined as maternal weight in kilograms (kg)/height^2^ (meters). WHO classification was used for the principal cut-off points for adult underweight, normal range, overweight and obesity: i.e. underweight <18.50 kg/m^2^, normal range 18.50–24.99 kg/m^2^, overweight ≥25.00 kg/m^2^, and obesity ≥30.00 kg/m^2^.

*Work description*. Participants were asked at Q1 about their primary employment status prior to the recent pregnancy with the response alternatives: ‘gainfully employed’, ‘student’, ‘maternity leave’, ‘unemployed’ and ‘on sick leave’. They were also asked what kind of description that defined their job the best (with the possibility to give more than one option). The options were ‘mainly sitting’, ‘physically active’, alternatively sitting/physically active’, ‘physically challenging’, ‘physically easy’, ‘alternatively physically challenging/easy’, ‘mentally challenging’, ‘mentally not challenging’, ‘alternatively mentally challenging/no challenging’, ‘intellectually stimulating’, ‘intellectually not stimulating’ and alternatively intellectually stimulating/not stimulating’.

*Hypermobility*. Women were asked at Q1 if they had previously been diagnosed as having hypermobile joints with the response alternatives ‘yes’ or ‘no’. Additionally, they were asked if they had any family member that had been diagnosed as hypermobile and whether they experienced themselves as hypermobile. The response alternatives were ‘yes’, ‘no’ and ‘do not know’.

*Self-rated health (SRH)*. The women were asked to assess their present health status at Q1, Q2 and Q3. A five category response options was used with the options: ‘very good’, ‘quite good’, ‘fair’, ‘quite poor’ and ‘poor’.

Baseline variables such as pre- and end-pregnancy weight, height, hypermobility, SRH during pregnancy, level of education and work description were obtained from the first questionnaire (Q1). Current weight and SRH at 6 months and 12 months postpartum were obtained from both Q2 and Q3 respectively. BMI was calculated from the self-reported measures at all three measured time points.

### Statistical methods

Descriptive statistics was used to investigate sick leave and healthcare utilisation in women with ‘recurrent’ or ‘continuous’ PLBP/PGP at approximately 12 months postpartum. The data were analysed through calculation of means and standard deviations (SD) for parametric data. The independent-samples *t* test was used to test for difference between respondents and non-respondents when possible. Pearson’s Chi-square test was used when testing for difference between respondents and non-respondents in regard to categorical data. For data not normally distributed median and interquartile range (IQR) was used.

Statistical significance was set at *p* < 0.05 for all analyses. IBM SPSS Statistics 20 software package was used.

### Ethical approval

The study was approved by the Ethics Committee at the Umeå University (Dnr 01–335).

## Results

Individuals who responded to Q3 (*N =* 176) were classified into three groups: ‘no’ pain (*N =* 34, 19.3 %), ‘recurrent’ pain (*N =* 115, 65.3 %), and ‘continuous’ pain (*N =* 27, 15.3 %). A detailed description of characteristics of the cohort has been previously presented [1]. Most participants had 2 children (38.1 %) and 36.9 % had one child. The vast majority were married or cohabiting (96.0 %) when responding to Q3. One hundred and fifty-nine (90.3 %) of the women were non-smokers and 170 (96.6 %) had at least achieved a high school education. Six out of 10 women (*N =* 107, 60.8 %) reported physical activity on a regular basis and assessed their health status to be ‘quite good’ (*N =* 84, 48.0 %) to ‘very good’ (*N =* 27, 15.4 %). Mean BMI at Q3 was 25.4 (SD 4.6) kg/m^2^. Relationship satisfaction was considered stable. No statistically significant differences between the subgroups were found, except for smoking, where the ‘continuous’ pain group included significant more smokers than the ‘recurrent’ pain group. Further description of the cohort can be found in Table 1.

**Table 1:** Descriptive information of the study group

	No pain^a^	Recurrent pain^b^	Continuous pain^c^	Total
	*N* = 34	*N* = 115	*N* = 27	*N* = 176
BMI at Q3, mean (SD)	26.23 (4.3)	25.40 (4.8)	24.22 (3.7)	25.37 (4.6)
Pre-pregnancy Q1 (SD)	26.30 (4.9)	25.13 (4.7)	24.33 (4.2)	25.24 (4.7)
< 18.50, n (%)	1 (2.9)	4 (3.5)	-	5 (2.9)
18.50–24.99, n (%)	13 (38.2)	60 (53.1)	16 (59.3)	89 (51.1)
≥ 25.00, n (%)	15 (44.1)	33 (29.2)	8 (29.6)	56 (32.2)
≥ 30.00, n (%)	5 (14.7)	16 (14.2)	3 (11.1)	24 (13.8)
End-pregnancy Q1 (SD)	32.12 (5.3)	30.38 (4.8)	30.15 (4.5)	30.68 (4.9)
Underweight, n (%)	-	-	-	-
Normal range, n (%)	3 (8.8)	14 (12.4)	2 (7.4)	19 (10.9)
Overweight, n (%)	7 (20.6)	41 (36.3)	12 (44.4)	60 (34.5)
Obesity, n (%)	24 (70.6)	58 (51.3)	13 (48.1)	95 (54.6)
At 6 month post-partum Q2 (SD)	26.57 (4.7)	25.37 (4.49)	24.98 (3.9)	25.54 (4.4)
< 18.50 (underweight), n (%)	1 (3.0)	3 (2.8)	-	4 (2.4)
18.50–24.99 (normal range), n (%)	11 (33.3)	56 (51.4)	15 (55.6)	82 (48.5)
≥ 25.00 (overweight), n (%)	16 (48.5)	35 (32.1)	8 (29.6)	59 (34.9)
≥ 30.00 (obesity), n (%)	5 (15.2)	15 (13.8)	4 (14.8)	24 (14.2)
At 14 months post-partum Q3 (SD)	26.23 (4.3)	25.40 (4.8)	24.22 (3.7)	25.37 (4.5)
< 18.50 (underweight), n (%)	1 (3.1)	2 (1.8)	-	3 (1.8)
18.50–24.99 (normal range), n (%)	10 (31.3)	58 (52.3)	19 (70.4)	87 (51.2)
≥ 25.00 (overweight), n (%)	16 (50.0)	34 (30.6)	6 (22.2)	56 (32.9)
≥ 30.00 (obesity), n (%)	5 (15.6)	17 (15.3)	2 (7.4)	24 (14.1)
Employment status Q1				
Gainfully employed	24 (70.6)	77 (68.1)	19 (70.4)	120 (69.0)
Student	4 (11.8)	10 (8.8)	1 (3.7)	15 (8.6)
Maternity leave	3 (8.8)	11 (9.7)	3 (11.1)	17 (9.8)
Unemployed	1 (2.9)	3 (2.7)	-	4 (2.3)
On sick-leave	2 (5.9)	12 (10.6)	4 (14.8)	18 (10.3)
Work description Q1				
Mainly sitting	8 (23.5)	16 (14.0)	6 (33.3)	30 (17.2)
Physical active	18 (52.9)	58 (50.9)	10 (38.5)	89 (49.4)
Alternate sitting/physically active	8 (23.5)	40 (35.1)	10 (38.5)	58 (33.3)
Physically challenging	8 (24.2)	38 (33.9)	8 (33.3)	54 (32.0)
Physically easy	13 (39.4)	33 (29.5)	6 (25.0)	52 (30.8)
Alternate physically challenging/easy	12 (36.4)	41 (36.6)	10 (41.7)	63 (37.3)
Mentally challenging	5 (16.7)	35 (32.1)	8 (32.0)	48 (29.3)
Mentally not challenging	11 (36.7)	17 (15.6)	4 (16.0)	32 (19.5)
Alternate mentally challenging/not challenging	14 (46.7)	57 (52.3)	13 (52.0)	84 (51.2)
Intellectually stimulating	14 (42.4)	56 (50.5)	8 (33.3)	78 (46.4)
Intellectually not stimulating	5 (15.2)	10 (9.0)	4 (16.7)	19 (11.3)
Alternate intellectually stimulating/not stimulating	14 (42.4)	45 (40.5)	12 (50.0)	71 (42.3)
Hypermobility diagnosis Q1				
Yes	7 (20.6)	23 (20.5)	9 (33.3)	39 (22.5)
No	27 (79.4)	89 (79.5)	18 (66.7)	134 (77.5)
Number of visits to healthcare providers the past 6 months at Q3, median (IQR)				
Acupuncture	-	4 (4–4)	1 (1–1)	2.5 (1–2.5)
Chiropractic	-	5 (2–5)	1 (0–1)	2 (1–6)
Medical doctor	-	1 (1–1.25)	2 (1–2.5)	1 (1–2)
Naprapathy	-	3 (1–3)	5.5 (3–5.5)	3 (2–6.5)
Physiotherapy	-	3 (1–5.25)	7 (1–12)	3 (1–7.5)
Other (including visits to midwife)	-	2 (1–2)	1 (1–1)	1 (1–2.5)

Numbers in parenthesis are percentage unless otherwise specified

^a^‘No pain’ denotes respondents reporting remission of LBP/PGP at Q3

^b^‘Recurrent pain’ denotes respondents reporting recurrent LBP/PGP at approximately 14 months post-partum at Q3

^c^‘Continuous pain’ denotes respondents reporting continuous LBP/PGP at approximately 14 months post-partum at Q3

### Sick leave

**Fig. 2:**
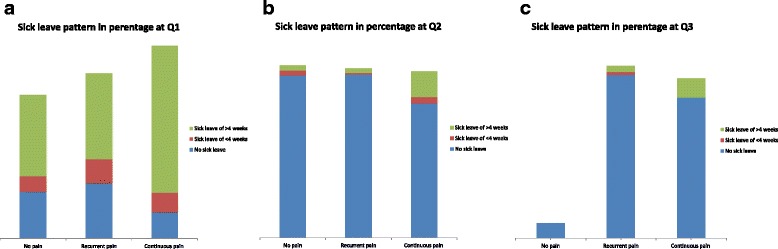
**a**, **b** and **c** No sick leave and sick leave for more or less than 14 days for each subgroup at Q1, Q2 and Q3

The vast majority of women (92.4 %) who responded at Q3 reported no sick leave during the past 6 months. However, Table 2 demonstrates that women with ‘continuous’ pain at Q3 had been on sick leave to a higher extent at all measure points compared to women reporting ‘recurrent’ pain at Q3. Additionally, women with ‘recurrent’ and ‘continuous’ pain reporting sick leave at Q1, Q2 and Q3 had been so on a full-time basis. Women with ‘recurrent’ pain reported long-term sick leave of more than 4 weeks to a higher extent compared to the ‘no’ pain group at Q1 and the ‘continuous’ pain group demonstrated a higher degree of long-term sick leave at both Q1 and Q2 compared to the ‘no’ pain group (Fig. 2a, b and c). In addition, women with ‘continuous’ pain demonstrated more long-term sick leave compared to women with ‘recurrent’ pain at all three measured time points.

**Table 2:** Sick leave and degree of sick leave due to PLBP/PGP at Q1, Q2 and Q3 with 95 % confidence intervals. Test for difference between respondents and non-respondents (Pearson's Chi test)

	No pain^a^		Recurrent pain^b^		Continuous pain^c^		Non-respondents	
	Number of subjects	95 % CI^e^	Number of subjects	95 % CI^e^	Number of subjects	95 % CI^e^	Number of subjects	*p*-value^d^
	34		115		27		24	
Sick leave Q1, N (%)								
Yes	16 (72.7)	1.07–1.47	61 (67.8)	1.22–1.42	22 (84.6)	1.01–1.30	10 (58.8)	0.271
No	6 (27.3)		29 (32.2)		4 (15.4)		7 (41.2)	
Sick leave Q2, N (%)								
Yes	2 (5.9)	1.86–2.02	5 (4.4)	1.92–1.99	6 (22.2)	1.61–1.95	1 (4.2)	0.554
No	32 (94.1)		108 (94.7)		21 (77.8)		23 (95.8)	
Sick leave Q3, N (%)								
Yes	-	-	6 (5.3)	1.91–1.99	5 (18.5)	1.66–1.97	-	-
No	3 (8.8)		108 (94.7)		22 (81.5)		-	
Degree of sick leave Q1, N (%)								
Full-time	8 (47.1)	1.29–2.01	50 (73.5)	1.20–1.51	19 (82.6)	1.00–1.61	10 (90.9)	0.276
Part-time	7 (41.2)		12 (17.6)		1 (4.3)		-	
Both full- and part-time sick leave	2 (11.8)		6 (8.8)		3 (13.0)		1 (9.1)	
Degree of sick leave Q2, N (%)								
Full-time	2 (100)	-	4 (80.0)	0.64–1.76	6 (100)	-	1 (100.0)	0.773
Part-time	-		1 (20.0)		-			
Degree of sick leave Q3, N (%)								
Full-time	-	-	4 (66.7)	0.79–1.88	3 (60.0)	0.72–2.08	-	-
Part-time	-		2 (33.3)		2 (40.0)		-	

^a^‘No pain’ denotes respondents reporting remission of LBP/PGP at Q3

^b^‘Recurrent pain’ denotes respondents reporting recurrent LBP/PGP at approximately 14 months after pregnancy at Q3

^c^‘Continuous pain’ denotes respondents reporting continuous LBP/PGP at approximately 14 months after pregnancy at Q3

^d^Respondents vs.non- respondents at Q3

^e^95 % confidence interval (CI) regarding sick leave yes/no and degree of sick leave at Q1, Q2 and Q3

Significance test *p* < 0.05

### Healthcare utilisation

The majority of women reporting ‘recurrent’ or ‘continuous’ pain at Q3 had not sought any healthcare services during the past 6 months (*N* = 91, 64.1 %). However, 59.3 % (*N* = 16) women with ‘continuous’ pain did report that they had sought healthcare services the past 6 months compared to 30.4 % (*N* = 35) of women with ‘recurrent’ pain at Q3.

**Fig. 3:**
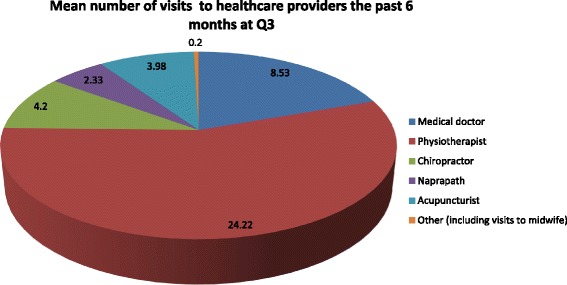
Number of visits to healthcare providers the past 6 months at Q3

The most sought healthcare service was physical therapy followed by medical doctor (MD) consultation, acupuncture, chiropractic and naprapathic treatment for both groups of women reporting pain at Q3. Other types of treatments that were reported consisted of stretch exercises, ultrasound treatment, Reiki healing, osteopathic treatment, exercise programs and massage therapy (including consultation with a midwife) (Fig. 3).

No treatment alternative was perceived as being more successful than any of the alternatives listed i.e. having a good perceived effect on symptoms.

## Discussion

### Sick leave

The first objective of this study was to investigate the prevalence, pattern and degree of sick leave in women reporting persistent PLBP/PGP at 12 months postpartum. The findings revealed that most women had no sick leave during the past 6 months before responding to Q3 despite the majority of the women still reported ‘recurrent’ or ‘continuous’ pain. Nevertheless, women with ‘continuous’ pain at Q3 had been on sick leave to a higher extent and demonstrated more long-term sick leave at all measure points compared to women reporting ‘recurrent’ pain at Q3.

This cohort had complete baseline information on all subjects and is to the best of the authors’ knowledge the first study that has investigated long-term prevalence, pattern and degree of sick leave among women reporting PLBP/PGP during pregnancy. Studies on LBP in the general population show previously reported sick leave and episodes of LBP are predictors of poor outcome and thus persistence of symptoms and delayed recovery rate [8, 26–28]. Thus, there may be reason to believe that sick leave due to persistent PLBP/PGP during and shortly after pregnancy is a risk factor to consider in terms of long-term problems postpartum. Other potential risk factors known to influence poor outcome/persistency of symptoms in regard both LBP and PGP are, but not limited to, age, marital status, duration of symptoms, psychological stress, low levels of physical activity, heavy physical work, high BMI, hypermobility, level of education and reduced SRH [1, 8, 11, 23, 28–33].

It is well established that thoughts, feelings and beliefs of an individual have significant impact on LBP [34]. In addition, individual coping strategies are considered important contributors to future disability in regard to LBP and psychosocial factors appear to exacerbate the clinical component of pain [34, 35]. The presence of emotional distress during pregnancy is shown to be associated with poor outcome [36] and catastrophizing and disability during pregnancy have been shown to increase the risk of postpartum lumbopelvic pain [37]. Albeit the questionnaire used in this study did not include any psychosocial factors with the exception of relationship satisfaction and family situation, reduced SRH has shown to negatively influence the recovery of LBP [29]. In addition, we have demonstrated in a previous paper that women with persistent PLBP/PGP postpartum and ‘continuous’ pain reported less favourable health status compared to women with ‘recurrent’ pain [1]. Thus, previous sick leave and poorer SRH could contribute to why women reporting ‘continuous’ pain at Q1 also reported a higher degree of long-term sick leave at Q2 and Q3.

### Healthcare utilisation

**Fig. 4:**
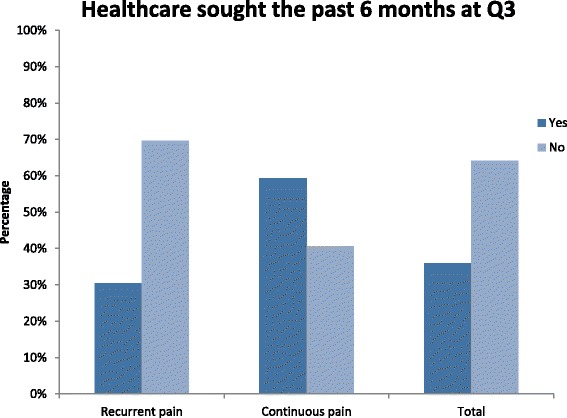
Healthcare sought the past 6 months at Q3 in women reporting ‘recurrent’ pain, ‘continuous’ pain and total number of women

Somewhat surprisingly this study shows that women with persistent PLBP/PGP at Q3 had not sought any healthcare service during the past 6 months (Fig. 4). In a previous study, we demonstrated that women with ‘continuous’ pain did report statistically higher pain intensity compared to women with ‘recurrent’ pain [1]. However, the most important reason for seeking healthcare in the general LBP population has shown to be high levels of disability and not pain itself [38], which could partly explain why most women with persistent PLBP/PGP do not seek care. Most participants may still be on parental leave hence not affecting their work performance or prevent them from taking care of their infant. Another reason could be that there are no effective treatments for this condition today [39–41] and many women with persistent PLBP/PGP postpartum may feel brushed aside by healthcare professionals due to lack of information regarding persistent PLBP/PGP and/or believing themselves that symptoms will subside with time [42, 43]. However, those who seek healthcare appear to suffer from more severe back pain with more functional limitation and demonstrate poorer health-related-quality-of-life scores compared to non-healthcare seekers [44], which can also be a possible explanation to our findings. In a study of the same cohort, we have demonstrated that women with ‘continuous’ pain have a higher likelihood of poorer SRH compared to women with ‘recurrent’ pain [1]. These prior findings could therefore explain why women with ‘continuous’ pain sought care to a higher extent compared to women with ‘recurrent’ pain at Q3 (Fig. 4).

In this study physiotherapy was the most common sought therapy the past 6 months at Q2 and Q3 among women with ‘recurrent’ and ‘continuous’ pain. Physiotherapy in Sweden is well integrated in the public healthcare system and referral from MD is most often not necessary. In addition, there is a reduced patient’s fee, subsidized by the county council, when visiting a public or private practicing physiotherapist. However, a systemic review from 2003, investigating the effectiveness of physiotherapy in women with PLBP/PGP, is inconclusive regarding its effectiveness [45]. The majority of women in this study did report at Q3 that physiotherapy had ‘some effect’ on their symptoms. However, data is lacking in regard to what kind of treatment was received during the physiotherapy visits.

Stuge et al. [22] examine the long-term effect of physiotherapy with core-stabilizing exercises compared to physiotherapy without core-stabilizing exercises (control group) in women with persistent PGP postpartum. Their study shows a significant difference between the treatment group and the control group where low levels of pain and disability are maintained in the treatment group 2 years postpartum [22]. However, their control group show a significant improvement in functional status from 1 to 2 years after delivery. Conversely, a review article by Ferreira et al. [46] conclude that more high quality randomized clinical trials are needed as evidence regarding effectiveness of physiotherapy in regard to pregnancy related LBP and/or PGP is inconclusive.

Consultation with a medical doctor was the next most commonly sought health service and was also somewhat expected, as MDs are the only health professionals licensed in Sweden to prescribe analgesics (with the exception of dentists and veterinarians). Other aspects that could have affected the number of visits to the MD are that a person on sick leave for more than 1 week needs a sick leave certificate issued by a MD and every fourth person in Sweden believes that a referral issued by a MD is necessary to see a physiotherapist [47]. The majority of women did not find that analgesics had a good effect on their symptoms. Moreover, it is difficult to determine the effect a specific painkiller had on reported symptoms, as no information was available in respect to what kind of painkillers were used. For instance, recent research shows that paracetamol does not have any effect on LBP symptoms [48, 49]. In addition, paracetamol does not show any effect on pain, disability, function, global system change, sleep quality or quality of life, casting doubt concerning the universal endorsement of the use of paracetamol for LBP [48, 49]; therefore, there are reasons to believe that the same is true for PLBP/PGP.

Acupuncture treatment was reported to have ‘some’ to ‘good’ effect on symptoms in women with ‘recurrent’ and ‘continuous’ pain at both Q2 and Q3. A study by Elden et al. [50] show that acupuncture has some improvement on performing daily activities; however, acupuncture has no effect on PGP symptoms or the degree of sick leave compared to sham treatment. Unfortunately, information was lacking in regard to the perceived effect of chiropractic and naprapathy on symptoms. To the best of the authors’ knowledge, there is a lack of evidence in regard the effectiveness of spinal manipulative therapy (SMT) in women with persistent PLBP/PGP postpartum [39]. Nevertheless, there are some studies that indicate that SMT may decrease pain symptoms as well as having a positive effect on function in women experiencing PLBP/PGP during pregnancy [20, 41, 51]. Other treatment consisted of several different treatments (i.e. stretch exercises, ultrasound treatment, Reiki healing, osteopathic treatment, exercise programs and massage therapy) making it impossible to say what kind of treatment had a good perceived effect on symptoms.

In concurrence with this study, a recently published study demonstrate that individuals with self-reported musculoskeletal pain during the past two weeks show a statistically significant increase in utilisation of general healthcare services [18]. In addition, individuals with primary pain sites from the neck and low back are more likely to seek care from a physiotherapist or chiropractor [18].

### Methodological considerations

There are some methodological considerations that need to be addressed in this study. This study commenced in 2002 and at that time no international definition of PGP was available [4]. Instead, pain drawings were used to indicate the location of pain in the pelvic/lumbar area [23]. As a result of the introduction of international definitions and that pain sites of PGP often correlate with common pain location of LBP, some of the cases in our study might be misclassified. However, the lifetime prevalence of LBP is considered stable [52], while pelvic girdle pain increase during pregnancy [33]. In addition, we have previously demonstrated that most women indicated a ‘mixed pain location’ (back and front of the lumbopelvic area), indicating a strong likelihood that the pain drawings in this study are mostly related to PGP [1]. There also appear to be an increased risk of persistent PGP in women experiencing both LBP and PGP during pregnancy [2, 53]. Thus, a misclassification of non-cases would result in an underestimation of associations.

In this study, sick leave was self-reported and could thus be considered a limitation. However, self-reported sick leave is shown to have good agreement with recorded information on number of sick-days, thus retrospectively collected self-reported numbers of sick-days can be useful when registered data are not available [54]. There is also a risk that sick leave was underreported in this study. According to the Swedish Parental Leave Act [55], both mothers and fathers can be on parental leave until the child is 18 months old making it plausible that many women might still have been on parental leave at Q3, thus not bothering to report sick leave to the Swedish Social Insurance Agency as long as their problems did not affect their ability to take care of their infant.

## Conclusion

In summary, the main findings in this cohort study was that the majority of women did neither report sick leave nor sought any healthcare services during the past 6 months at Q3 despite reporting ‘recurrent’ or ‘continuous’ pain. However, women with more pronounced problems (‘continuous’ pain) did report a higher prevalence and degree of sick leave and healthcare seeking behaviour compared to women with less pronounced problems (‘recurrent’ pain). Women with more pronounced problems might constitute a specific subgroup of patients with persistent PGP where the long-term outcome is less favourable. More research is needed in regard to sick leave and healthcare utilisation due to persistent PGP postpartum, powered to determine associations between previously reported poor prognostic factors and sick leave postpartum as well as care-seeking behaviour.

Clinicians need to be attentive that PLBP/PGP may not be transient for some women; instead some will become chronic in nature. Consequently, pregnant women may need to be screened early in pregnancy as well after childbirth facilitating early and customized treatment intervention for PLBP/PGP, consequently reducing individual suffering and societal cost as well as decreasing the risk of transition into chronicity. More clinical research is needed to evaluate the possible effective treatments for this condition both during pregnancy and postpartum.
